# Development of an HPV 16 rapid test founded in user-centered design with primary care clinicians

**DOI:** 10.1039/d5ay01621e

**Published:** 2025-11-25

**Authors:** Luke P. Brennan, Francesca Hamacher, Layla A. Claure, Ana L. Claure Dips, Sathveka Sembian, Lara N. Balian, Andrew D. Piepho, Aaron C. Ermel, Jacqueline C. Linnes, Natalia M. Rodriguez

**Affiliations:** a Weldon School of Biomedical Engineering, Purdue University West Lafayette 47906 IN USA jlinnes@purdue.edu nmrodriguez@purdue.edu lukebrennan002@gmail.com anaclauredips@outlook.com sathveka@gmail.com piepho@purdue.edu rodrignm@purdue.edu; b Department of Public Health, College of Health and Human Sciences, Purdue University West Lafayette 47906 IN USA claurelayla@gmail.com lbalian@purdue.edu; c Indiana University School of Medicine, Indiana University Indianapolis IN USA aermel@iu.edu; d Agricultural and Biological Engineering, Purdue University West Lafayette 47906 IN USA fchiara72@gmail.com; e Cancer Prevention and Control Program, Indiana University Simon Comprehensive Cancer Center Indianapolis 46202 IN USA

## Abstract

Despite effective screening modalities, cervical cancer remains a leading cause of cancer-related death among women in the United States aged 20 to 39 years old, and incidence is rising in women aged 30–44 years old. Up to 25% of patients who are screened for cervical cancer by testing for human papillomavirus (HPV) do not receive necessary follow-up care with current laboratory-based testing. Applying a user-centered design approach, we surveyed and interviewed practicing clinicians to establish the use case, value proposition, and user requirements of a cervical cancer screening test for use in Indiana, USA. Insights from these stakeholders directly informed design specifications for a point-of-care HPV test capable of providing same-visit results to improve patient follow-up and retention. Guided by these requirements, we designed an isothermal nucleic acid amplification platform suitable for outpatient clinics. The test accepts swabbed endocervical cells, amplifies HPV16 L1 DNA *via* recombinase polymerase amplification, and provides results within 40 minutes on a lateral flow assay. Further, the test achieves a clinically relevant limit of detection of 1000 HPV 16 copies per reaction and verifies swabbing technique and test operation with a sample adequacy control. The test operation was designed for a minimally-trained user and decreases time-sensitive steps that would interfere with clinical flow. By integrating clinician input to inform development decisions, our device is uniquely tailored to meet the context-specific needs of primary care clinics. This work exemplifies how user-centered design can yield novel diagnostic technologies with greater clinical impact and adoption potential.

## Abstract 2

Clinician-informed nucleic acid test for cervical cancer screening to facilitate same-visit results to improve follow-up care.

## Graphical abstract

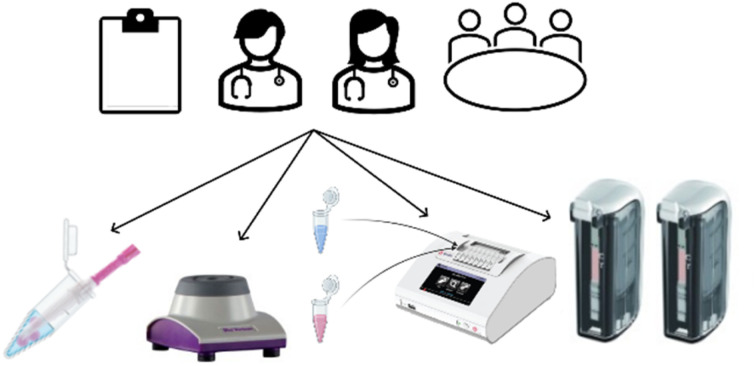

## Background

Point-of-care tests (POCTs) hold the promise of increasing access to diagnostic testing and expediting care, yet uptake and adoption are a struggle for even effective POCTs. POCTs are often not lower cost to implement for patients or clinics, and many clinicians fear compromising the high perceived accuracy of laboratory tests by using POCTs.^1,2^ Further, POCTs are often underpromoted in guidelines or institutions, and patients may not know they are an option. This makes it imperative that a POCT demonstrate value clearly and quickly to clinical adopters. POCT value is often obscured by a mismatch between the test's design characteristics and the role the healthcare system expects the test to play. In response, our group used a user-centered design approach to develop an HPV POCT for cervical cancer screening.

In the United States, 60% of cervical cancers occur in individuals who are not up-to-date with screening^3,4^ and 25% of eligible Americans are behind in screening.^5^ This is largely because existing cervical cancer screening methods require a preventive healthcare visit, an invasive sampling procedure, and reliable methods to contact patients with their laboratory results. Based on data from 2016–2019, an individual's educational level, race, insurance status, and being foreign-born with less than 10 years in the US contribute to inequities in cervical cancer screening.^6–8^ Even when patients are successfully screened, an estimated 25% fail to receive proper follow-up, putting them at higher risk of developing cancer unchecked.^8–10^

The well-adopted screening strategy of primary HPV testing^11–14^ obtains an endocervical sample that is then used to detect DNA or RNA from high-risk HPV (hrHPV), the virus that causes most cervical cancers, to determine next steps. Mounting evidence demonstrates that patients can reliably collect their own sample for cervical cancer screening, and in 2024 the FDA approved in-clinic self-sampling with specified collection devices and hrHPV laboratory tests.^15–19^ These developments allow for in-clinic testing, mail-in testing, and at-home testing, which may improve uptake in under-screened populations. We aimed to assess whether clinicians would be willing to make use of these developments in screening methods, and if so, how to design an hrHPV test to deliver the value they hope to receive.

In this study, clinicians who conduct cervical cancer screening in Indiana, USA, were interviewed and surveyed. Indiana was chosen as a case study because, with a single set of healthcare laws, regulations, patient population, insurance providers, and a patient population pooled together in national data, it can be reasonably analyzed as a cohesive analytical unit. Yet, it remains large enough to hold public health significance. Indiana ranks among the top ten US states for cervical cancer mortality, with the age-adjusted death rate increasing in the past 5 years.^20^ The state's screening prevalence among women aged 21–65 years is 76%.^21^ This matches the US screening prevalence of 76% in 2023,^22^ and reflects the racial and socioeconomic disparities seen in the US as a whole.^23^ As the home of the researchers, Indiana also presented an accessible clinician population with demonstrated need for improved cervical cancer control.^20^

Clinicians were asked which screening methods they were interested in adopting, and what characteristics they would need in an hrHPV screening test for adoption. Respondents' feedback informed the intended use, user requirements, and value proposition of such a test. Uniquely, this input was solicited before test design, allowing the findings to be translated into design specifications and thereby govern the inception and development of the test to fit its exact use case. These considerations included accepting provider-collected rather than patient-collected swabs, an interest in confirming negative results with a laboratory test, and a strong preference to explain results in person. The product is a sample-to-answer nucleic acid amplification test (NAAT) for hrHPV using primers adapted from a clinically validated primer set. The testing workflow starts with sample from an FDA-approved cervicovaginal swab, amplifies HPV 16 DNA alongside human DNA as a sample adequacy control, and develops a lateral flow assay (LFA) in an enclosed housing to prevent workspace contamination. The test is designed to be performed and interpreted in an outpatient clinic in the United States during the patient's visit. As a proof of concept, our test accurately detects HPV 16 from cell lysates on contrived cellular swabs. Notably, the test is uniquely suitable to its role as a ‘rule out’ test since the sample adequacy control identifies true negatives among invalids and detects more sources of test error than an internal control or a standard lateral flow control line. Not only should this limit the need to verify negative results with laboratory testing, but it also highlights a way to address a longstanding rapid test issue of invalid results being reported as false negatives to lower clinical sensitivity.^24^

This hrHPV test was created using a design method that established and prioritized stakeholder practices, attitudes, and needs very early in the design process. This approach yielded a point-of-care testing process that performs sample-to-answer nucleic acid amplification testing in 40 minutes with a sample adequacy control. The process includes no technically demanding user steps, and requires no custom instrumentation.

## Methods

### User-centered study design, distribution, and analysis

The user-centered survey design has been described in prior works.^1,25,26^ Briefly, in 2021, 224 eligible clinicians (nurse practitioners and physicians in family medicine, OB/GYN, or internal medicine specialties) were voluntarily surveyed using the survey software Qualtrics (Utah, USA), and 20 clinicians were interviewed. Eligibility criteria included having conducted cervical cancer screening on average-risk females aged 21–65 years in Indiana, US, in the previous month. Interviewees and survey respondents were provided with information about the purpose and scope of the survey, and consented with digital signature or verbal affirmation to have their deidentified responses published for research purposes.

Questionnaires queried interviewees and survey respondents about practices, knowledge, and attitudes regarding current cervical cancer screening methods, potential screening methods, and requirements of an adoptable test. Validated items from other surveys were adapted whenever possible.^27–32^ Questionnaire items addressed in the data presented herein are included in the supplemental materials. This study was approved by the Institutional Review Board at Purdue University (protocols: IRB-2019-132; IRB-2021-12; IRB-2021-617).

Descriptive analyses were performed using SPSS v28, and desired test characteristics were weighted 1 to 5 in increasing levels of adoptability and summed across respondents. Interviews were analyzed through qualitative thematic analysis of potential use cases and user requirements for an hrHPV test.

### hrHPV test development

#### Materials

For cell sampling experiments, Viba Brush (Rovers, WA, USA), nylon-flocked swab (MedSchenker, NJ, USA), and polyester-flocked swab (Puritan, ME, USA) were purchased for use with 2 µL Eppendorf Protein LoBind tubes (Hamburg, Germany).

Plasmids were purchased from GenScript (NJ, USA), and primers and probes were ordered from Integrated DNA Technologies (IDT) (Iowa, USA). C-33A cells, an HPV-negative human cervical epithelial cell line, were purchased, and CaSki cells, an HPV 16-positive human cervical epithelial cell line, were kindly provided by Dr Sulma Mohammed (ATCC Products HTB-31 and CRL-1550, Manassas, VA). Eagle's Minimum Essential Medium (EMEM) and Roswell Park Memorial Institute Medium (RPMI), each supplemented with 10% fetal bovine serum (FBS) and 1% antibiotic–antimycotic solution, were purchased from Thermofisher Scientific (Waltham, MA). Recombinase polymerase amplification (RPA) basic kits were purchased from TwistDx (Cambridge, UK), and the T16-ISO instrument used as an incubator/imager was purchased from Axxin (Fairfield, Australia). RPA reactions were vortexed using Heathrow Scientific's Mini Vortexer (Illinois, USA). vWR Microcaps TLC Spotting Capillaries were used to transfer RPA reaction fluid and sample volume (PA, USA). Ethylenediaminetetraacetic acid (EDTA) was purchased from Thermofisher Scientific (MA, USA). HybriDetect 2T LFAs with two test lines were purchased from Milenia Biotec (Gießen, Germany), and enclosed lateral flow assay cartridges were purchased from BioUstar (Nucleic Acid Probes Detection Devices product # U40003, Hangzhou, China). LFAs were scanned using an Epson Perfection V850 Pro scanner (Suwa, Japan).

#### Template DNA

pUC57 plasmids bearing the HPV 16 L1 gene (GenBank ID K02718) were used as DNA standards. For cellular DNA, HPV-negative C-33A and HPV 16-positive CaSki cells were incubated at 37 °C with 5% CO_2_ with EMEM and RPMI respectively, each supplemented with 10% FBS and 1% antibiotic antimycotic solution.

#### Making primers and probes

Gong *et al.* (2021) adapted the hrHPV polymerase chain reaction primer sets PGMY09/11 and GP5+/6+ primer sets for use in RPA to detect 13 of 14 hrHPV types.^33^ Of this primer pool, the PGMY11-B and GP6+ primers aligned to the HPV 16 gene (K02718.1) with the fewest mismatches, which is why they were chosen for this work. Using the PrimedRPA^34^ Python script, 100 candidates were generated for an HPV 16-specific probe, to be used with these primers. The candidates were then screened against the major HPV 16 gene sequence variants obtained from NCBI Gene using Geneious Prime 2023.2. IDT's Oligo Analyzer was used to assess hairpin structures and melting temperatures at the salt concentrations of RPA. Probe candidates with secondary structures that melted at or below the RPA running temperature of 39 °C were approved for further analysis. ThermoFisher's online Multiple Primer Analyzer tool was used to check candidate probes for homodimerizations and heterodimerizations against other sequences in the reaction. The analyzer tool interrogated interactions with a sensitivity level set to the second most rigorous setting. Candidates that showed significant dimerization, including >30% sequence matches and 5′ overhangs (which can be extended), were discarded. Candidates with significant binding (*E* value < 0.01) to any organism DNA commonly found in endocervical samples according to the package insert of a commercially available comparator test, Cobas HPV 4800,^35^ were discarded. The HPV 16 probe candidates were additionally screened against the L1 gene sequences of the other hrHPV types with the same threshold. The two remaining probe candidates were screened empirically using plasmid DNA containing the HPV 16 L1 gene. The candidate with the larger signal-to-noise ratio was selected for experiments.

Another 100 primer pairs and 100 probe candidates were generated *in silico* using PrimedRPA^34^ for the β-globin housekeeping gene (Gene ID: 3043), to be used as a signal for sample adequacy. The primer and probe candidates were screened in the same manner as the HPV 16 probe candidates. After hairpin, dimerization, and organism specificity screening were completed, the remaining candidates were four forward primers, two reverse primers, and two probes. These were then ordered from IDT for *in vitro* screening (IA, USA). Empiric testing with purified human gDNA for the largest signal-to-noise ratio identified the primer pair and probe used in future experiments.

The final primers and probes were end-labelled for detection on LFAs. The HPV 16 forward (F) primer was labelled with 5′ fluorescein to bind the color-producing gold nanoparticles, and the reverse (R) primer was unlabeled. The HPV 16 probe had a 3′ phosphorus group to prevent extension, and was 5′ labelled with biotin to bind the streptavidin at one of the LFA test lines. The β-globin primers and probes were labelled in the same way, except the probe was 5′ labelled with digoxigenin so that it bound the second test line instead of the first. Primer and probe sequences with labels are shown in Table S1.

#### Recombinase polymerase amplification (RPA) assays

RPA solutions were prepared according to the manufacturer's default protocol in a dedicated PCR clean area without modification to the assay buffers or additives. The protocol was modified to adjust the concentrations of the HPV 16 F and R primers and probe, which were used at a concentration of 480 nM each, while the β-globin F primer and probe concentrations were 96 nM each, and the R primer concentration was 48 nM. These concentrations were chosen based on iterative testing of different primer and probe combinations to optimize the real-time fluorescent signal-to-noise ratio generated for HPV16 and β-globin targets separately. Two and a half microliters (2.5 µL) of analyte DNA in water or 10% C-33A cell lysate, depending on the experiment, were split between two parallel 25 µL RPA reactions to prevent amplification interference between the hrHPV and β-globin assays. The parallel reactions were incubated in the Axxin T16-ISO incubator for 20 minutes at 37 °C, with samples vortexed for 5 seconds at 3200 rpm at 5 minutes into the reaction. An Axxin T16-ISO incubator with fluorescent reader was used for to monitor the reaction in real time as a process measure for the experimenters (data not reported). However, a simple heating block could likely be used for this step in clinical use. After incubation, reactions were then quenched with 1 µL of 250 mM EDTA.

#### Lateral flow assay (LFA) readout and analysis

LFAs with anti-FITC and anti-DIG test lines were placed upright in an Eppendorf tube. Two microliters (2.5 µL) of the RPA reaction volume was pipetted onto their sample pad, and they were rinsed with 100 µL of the included running buffer. In the reproducibility experiments, the RPA reaction volumes were run on BioUstar enclosed LFA cartridges to reproduce the workflow of an outpatient clinical setting. In both cases, the LFAs were removed from the buffer for analysis after 10 minutes.

LFAs were imaged on an Epson Perfection V850 pro scanner at 3200, and band quantification was conducted using a Matlab script from Holstein CA *et al.* (2014)^36^ which calculates the greyscale intensity of the test line as compared to a background area of the same LFA.

#### Limit of detection (LoD) for HPV 16 L1 gene using LFA

RPA reactions for LoD assays were performed with contrived samples of HPV 16 L1 gene plasmids diluted in water or 10% C-33A cell lysates in water. The plasmid DNA was added in 2 µLs of plasmid solution to test 10 000 to 0 copies per reaction for the LoD in water, and 25 000 to 0 copies per reaction for the LoD in lysates. LFAs were quantified by scanning them and analyzing the high-resolution digital images using the same Matlab software.^36^ A one-way ANOVA with Dunnett's post-test was performed to analyze differences between each concentration and the no-template-DNA control to estimate a LoD.

#### Sample collection and processing

To collect enough cells for our assay's LoD requiring 1000 genome copies per 25 µL reaction, a swab would need to collect 40 000 cells per sample in order to account for sample loss and dilution during sample preparation. Three (3) commercially available sampling devices were tested for sample cellularity by swabbing cells from culture flasks adapting a method from the literature,^37^ and Viba-Brush was selected for consistently satisfying this criterion (Fig. S2). Using Viba-Brush-collected samples, we found that vortexing 3× for 5 seconds at 3200 rpm with 10 seconds breaks was a simple and rapid method of cell lysis, which facilitated DNA detection with up to 20% RPA reaction volume as lysate. Several other methods including heat, detergents, acids, and bases were tested as alternative forms of cell lysis, but none could achieve acceptable lysis thresholds within the 10 minutes lysis threshold needed to return results within 40 minutes of sample collection.

#### Reproducibility of clinic-amenable sample testing process

The threshold test line intensity for positive and negative samples was determined as in Armbruster *et al.* (2008).^38^ Ten (10) C-33A and 10 CaSki cultured cell swabs were collected from 1 cm^2^ culture flask areas and processed as contrived samples as in Fig. 3. The ten paired samples were run in three batches so the processing time for each sample was similar to what it would be in a clinic setting. These were run alongside 5 negative controls (water) and 5 positive controls (25 000 copies of the HPV 16 plasmid in water) which were used to calculate a limit of detection. The LFA test line signal intensities were evaluated to be true/false positives and negatives based on a limit of detection calculated from the control samples based on the method by Armbruster *et al.* (2008).^38^

Amplicon sequence validation, primer and probe sequences with end-tags, calculations for expected sample cellularity, and a bill of materials can be found in the SI Primer and probe sequences section, Swabbing experiments section, and Sample cellularity calculations section.

## Results and discussion

### User-centered study design, distribution, and analysis

In total, 224 clinicians were surveyed and 20 were interviewed. The vast majority (82%) of clinicians surveyed would support the adoption of hrHPV testing at the point of care in their clinic. Support for adoption of self-sampling in clinic (50%) and at-home rapid testing (48%) was lower, with clinicians citing concerns about patients collecting reliable samples, administering the test properly, and losing interest in visiting their provider for other preventive care. Accordingly, the use case of our hrHPV test became performing routine screening of asymptomatic patients for cervical cancer through provider-collected, in-clinic hrHPV testing at primary care visits. These interviews identified key themes of clinician perspectives regarding hrHPV POC testing (Table 1). Interviewees saw great value in the ability to explain results to patients and arrange any necessary follow-up procedures face-to-face, in the same visit. This ability to provide same-visit results became the core value that our test provides for potential adopters and was the guiding aim for test development.

**Table 1:** Key themes of clinician perspectives on hrHPV POC testing

Theme	Representative quote
Delivering same-visit results is highly valuable	“Rapid information would give you another opportunity to educate patients and talk about follow-up testing if needed, follow-up management, and kind of the course and implications of HPV.” (Physician, obstetrician/gynecologist, female)
Test reliability is essential	“I think the key with screening for any type of cancer is that we have an accurate result, number one, an accurate test is performed.” (Physician, internal medicine, female)
Analytical performance is essential	“…[a blood lipid point of care test] at a previous clinic that I found it was conflicting with the lipids that I would draw through the lab. So I've never used it here. The ones that we do use are pretty on point.” (Nurse practitioner, family medicine, female)
Usability (of test and in clinic workflow) must be simple	“Sometimes we're short staffed with like medical assistants, you know, and they're also doing you know pregnancy tests and HIV and, you know, drawing blood and doing other things so it would have to be pretty, pretty simple.” (Nurse practitioner, planned parenthood, gender unspecified)
Compatibility with other STI testing is desirable	“I get a lot of patients who you're just here for their Pap, but they come back with trichomonas, they come back with chlamydia.” (Nurse practitioner, family medicine, female)
Uncertainty of sample adequacy	“The negative test I would always be a little questionable about because you just got to make sure it's done right and I'm not even saying that practitioners that do it are necessarily doing them right so there's always that user error that kind of worries me a little bit.” (Nurse practitioner, family medicine, female)
A simple readout of the test may be preferable for non-medical readers	“Pap results are very confusing. So to call someone and tell them they have low grade cervical changes, like that's very confusing to the non medical person. And so it's difficult as a provider to convey that in a message that then your staff is going to call a patient. So, you know, that's not ideal. I think positive HPV, negative HPV is very easy to understand. When you start talking about those cellular changes. That's not as easy.” (Physician, family med., female)
Cost comparability to current tests is important	“Cost would be important to be covered by insurance or cost to be comparable to testing that exists currently.” (Physician, family medicine, female)
Preference for in-clinic *vs.* at-home testing	“I think the most ideal situation would be the patient's in the office and you can kind of verbally go through it and in the room with them face to face. Because that way, I think you're still ensuring that you're getting like full comprehensive care. So not just the cervical cancer screening, but also that they're getting all their other care needs addressed.” (Physician, family medicine, female)

**Fig. 1:**
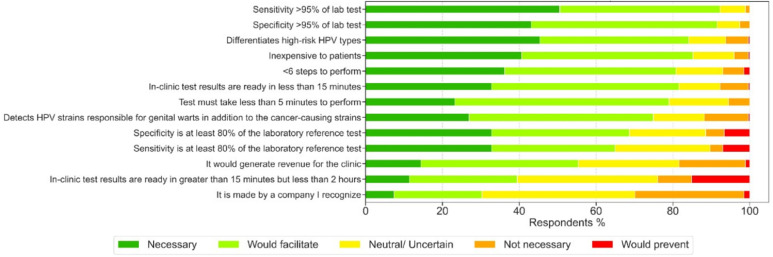
Ranked test characteristics for rapid test adoption.

Survey respondents were asked to report how several proposed test features would influence their adoption of an hrHPV rapid test. Combining and sorting these responses yielded a ranked list of preferred test features (Fig. 1), which was interpreted in conjunction with key themes from the interviews (Table 1). Analytical performance, as measured by sensitivity and specificity, was ranked as the most important factor in the interview data. Information about the infection, such as differentiating hrHPV types, was also very important to respondents, likely because the care algorithms for rapidly carcinogenic HPV types 16 and 18 differ from care algorithms for the other hrHPV types.^39–42^ Interview data balances these desires with a priority on communicating clear results to patients and among clinic staff. The expense to patients was the next consideration, which aligns with interviewee data indicating that testing should be covered by insurance, followed by three features related to test expediency and simplicity. Finally, interview data revealed that patients testing positive for hrHPV likely need testing for other sexually transmitted infections (STIs).

Other work by our group fleshes out the patient perspective towards new cervical cancer screening methods including self-sampling and running an HPV rapid test themselves.^43^ In-person recruiting and flyers were used to recruit women in Lake County, Indiana to answer questions about their healthcare and cervical cancer screening experiences. Survey respondents were female assigned at birth and between ages 21–65, while interviewees additionally needed to identify as Hispanic. Briefly, being Hispanic having Medicaid, and being uninsured were negatively associated with willingness to self-sample. Interviews identified concerns about remembering to perform the test and doing so correctly. Accordingly, while the surveyed patients see opportunities for increased comfort and less fear with self-sampling, the study concluded that vulnerable populations may not readily accept or adopt self-sampling at this time.

Surveyed clinicians were presented with 13 potential characteristics of a rapid hrHPV test and prompted “Assuming the test is FDA approved, how important is each characteristic for you to consider adopting rapid HPV testing for your patients (for tests performed at home or in clinic)”. When prompted for any additional test characteristics in a free response question, respondent answers fell into the categories of regulatory approval^3^ insurance coverage^5^ sample validity information^5^ accuracy^8^ ease of use/clarity of instructions (6/4, 10 total), interpretable results,^3^ and miscellaneous.^4^

Together, these user-centered findings from the survey and interviews provided guidance from the test's inception so that major design decisions rested on substantiated assumptions about the test's use case and value. Using this approach, data from users was paramount to the test's purpose, in addition to its appearance and operation. An HPV rapid test could serve as an at-home test, a patient-collected clinic test, or a provider-collected rapid test, but each of these uses requires different test capabilities. The test could also be used to prompt users to seek formal clinical testing, supplement lab testing to facilitate communication and follow-up care, or serve as an alternative to lab testing. The user-centered approach made it clear which testing circumstances and purposes would be most valuable to Indiana clinicians, and then established design specifications to fit this role. Information about executing user-centered design in healthcare can be found in Rodriguez *et al.* (2023)^44^ with case studies and theoretical frameworks. Patient and healthcare provider surveys from Townsend *et al.* (2014);^27^ Kim *et al.* (2016);^45^ Trope *et al.* (2009);^46^ Roland *et al.* (2013);^30^ Katz *et al.* (2017)^47^ were referenced in designing the provider survey and interview protocols. In this project, the user-centered design influenced the project in three significant ways:

First, clearly defining the test's use case, value proposition, and priority test characteristics provided specific criteria that were referenced when making design decisions from the start of the development process. For example, the test's value in facilitating same-visit results made time-to-result the most important design consideration, rather than cost or efficacy, when selecting methods for cell lysis, DNA amplification, and results readout. The test will likely be run by nurses or medical assistants who are already multitasking patient intake, other rapid tests, blood draws, and documentation. A ‘usable’ test in this context needed to minimize hands-on processing and incubation steps, especially incubation steps with precise timing requirements. Further, any test equipment needed to be simple and easily fixed or replaced, as the users were not responsible for maintaining rapid test instrumentation.

Second, clinicians were uncertain about trusting rapid tests for something as important as cancer screening. Many clinicians had used rapid tests that failed to report results, demonstrated poor analytical performance, or yielded false negative results with poorly collected samples. An effective rapid test for a cancer screening program needs to inspire confidence in its results. Our solution was to include a sample adequacy control, where the β-globin gene, present in every human cell, is detected alongside the hrHPV sample to distinguish between negative and invalid results. If the HPV 16 L1 gene is detected, the test is positive. If the HPV 16 L1 gene is not detected, detecting the β-globin gene indicates a true negative result as opposed to an invalid result. In an invalid result, a poorly collected sample or test processing error may be preventing HPV detection, even in an HPV-positive patient, and a new sample should be run on a new test. In future work, such sample adequacy controls may make rapid tests reliable enough to facilitate acceptance of rapid testing performed by patients themselves. After all, 70% of surveyed clinicians felt patient self-testing would improve cervical cancer screening.^1^

Third, clinicians seem to prefer interpreting test results themselves and explaining them to patients rather than having patients decipher the results on their own, which can lead to further confusion and distress. Clinician interviewees preferred a readout for ‘invalid result,’ ‘HPV 16 positive,’ another for ‘HPV 18 positive,’ and a third for ‘hrHPV other’ so they can explain and expedite the corresponding follow-up for each. On the other hand, if clinicians had opted for patients to run the tests themselves, they may have preferred a simpler readout of ‘invalid test,’ ‘positive, seek care,’ or ‘negative, repeat in 3–5 years.’

An excellent study by Nayak *et al.* (2019) highlights the utility of user-centered design from the first steps of the design process. After creating a working prototype the team uses the Fisher and Fisher IMB model for studying user behavior and the Honeycomb value model for user experience to elicit user experience and demand for a POCT for human immunodeficiency virus (HIV).^48^ This feedback allows the team to add features such as lights, switches, and a different vacuum actuation mechanism which will reduce variability in how users operate the test. However, the team also learned that many of their users have trouble drawing blood with lancets, want to use the device while inebriated, had a price point around half the cost of their current test, and would avoid intercourse with partners who were not willing to be tested. The team's prototype will require substantial changes or even a redesign to accommodate these revealed criteria. The team did diligent user-design work, however there is no replacement for input from users as early as possible in the design process.

### User requirements and design specifications

Clinician survey respondent data and key themes identified from interviews (Table 1) were used to create a set of user requirements to inform design specifications. Important criteria that were insufficiently addressed or quantified from clinician data were filled in using public material from the World Health Organization's target product profile,^49^ the Union for International Cancer Control,^50^ and other reports^51^ (Table 2). Some user requirements such as batch processing and the ability to easily add rapid test results into electronic health records, were not included in the table because evidence suggested that while it would facilitate adoption, it was not necessary in a minimum viable product.^49^ More user data about results reporting would need to be collected to inform these specifications. The test was compared against the design specifications to determine whether each was fulfilled, failed, or was not evaluated in the data presented in this report.

**Table 2:** User requirements and design specifications^c^

User requirements	Design specifications	Fulfilled
**Analytical performance**		
Perceived accuracy comparable to laboratory test standards	• LoD of 1000 HPV genome copies per sample^52^ maintained in the presence of cervicovaginal sample matrix, including with active microbial infection	Yes
	• Demonstrates a sensitivity^49^ and specificity >90% of reference test	Yes
Agreement with existing laboratory-based testing (HC2, cobas HPV 4800, Aptima)	Test demonstrates Cohen's kappa >0.41 with the clinic's reference test^53,54^	Untested
Genotyping of hrHPV types	Test differentiates hrHPV 16, 18 positivity from positivity for all other hrHPV types	No
Provide information about test validity (including sample adequacy and proper test function)	The test detects human DNA alongside hrHPV DNA. The detection of human DNA indicates that the sample was adequate for detection of hrHPV if present. This will be referred to as a sample adequacy control	Yes
If possible, provide information about other STI infections	The endocervical swab sample collection process for the hrHPV test described here:	
	• Does not prevent endocervical or vaginal swabbing for other tests in the same visit	Yes
	• The sample collected for hrHPV testing leaves at least half of the sample cell suspension volume in a medium free of chemical or molecular inhibitors	Yes

**Usability**		
Operate in outpatient clinics by a medical assistant or nurse	• Clinical Laboratory Improvement Amendments (CLIA) waived	Untested
	• Does not require reagents outside of the test kit^49^	Yes
	• <5 hours of focused device training is sufficient to perform the test without laboratory training^49^	Untested
Gives results quickly enough that a patient can be sampled and receive results in the same visit	• The test's results are available within 40 minutes^a^	Yes
Operable such that it does not significantly detract from other clinical duties of the test operator^a^	The test is operable such that:	
	• Test reagents are stable at room temperature for 15 minutes before sample addition	Yes
	• There are five or fewer user steps to perform^b^	No
	• The user does not have to watch or monitor any part of the test^a^	No
	• Test results can be communicated to a clinician in one sentence and recorded in an electronic health record in a standardized, searchable format^a^	Untested
Cannot put a cost burden on the clinic greater than existing laboratory-based testing after reimbursement^a^	Reimbursed by Medicaid and major insurers such that cost to clinic is equal to or less than laboratory testing.^a^ As an example, as of January 2025, the Indiana Medicaid fee-scheduled reimbursement for hrHPV testing was $35.09 USD^55^	Untested
Inexpensive to patients^a^^,^^b^	Total costs under USD 24 per test to the patient after Medicare and insurance coverage^56,57^	Untested
Allow providers to track the hrHPV status of their patients^a^	hrHPV test results and test date can be integrated into existing electronic health record patient tracking systems	Untested

a From Indiana clinician interviews.

b From Indiana clinician survey.

c A comprehensive table is provided in Table S2.

Survey and interview data accounted for 50% of the design specifications as compared to the external reports. Survey data indicated how much HPV type information providers wanted at the point of care, while the interviews highlighted the benefit of a swab collection process that allows for the patient's swab to be used in other tests, usually for sexually transmitted infections (STIs). Interviews were also essential in establishing the competing demands on the intended user's time and attention, as well as defining criteria for ‘affordability’. These additional data allowed design specifications to be tailored to their intended use case, user, and proposed value far more than the publicly available reports listed above.

### Overview of the proposed hrHPV test process

The test designed here begins with a cervical swab using a device that could be used for a self-sampling device. This device was specifically chosen to align with the growing trend and recent FDA approval of self-sampling as a viable alternative to clinician-administered sampling in outpatient settings.^18,19,58^ The collected cells are then released into assay buffer, lysed, and added to reagents of a nucleic acid amplification reaction. After a short incubation at 39 °C to amplify the DNA using RPA, the amplified DNA products are visualized on an LFA for test interpretation. One test line detects HPV 16 DNA by capturing the tagged HPV 16-specific probe on the LFA only if amplified HPV 16 L1 gene DNA links the probe to the biotinylated primer, which binds to streptavidin-coated gold nanoparticles. The other line serves as a sample adequacy control by detecting the anti-digoxigenin probe for the human β-globin gene, which acts as a proxy for human DNA, using tagged primers and probes. In this way, an HPV 16-negative sample would have signal at the sample adequacy control line but not the test line, whereas a positive sample would have signal on the test line, regardless of whether it develops signal at the sample adequacy control line.

Here we detect HPV 16 as proof-of-concept, however it is based on a primer set that can efficiently amplify 13 of the 14 hrHPV types^33^ which allows new hrHPV types to be detected by creating additional oligonucleotide probes readouts on the same LFA. Other rapid tests for HPV have been developed. The clinical guidelines of the US market dictate that nearly all the 14 hrHPV types be detected, narrowing the field of relevant tests. The commercial platform GeneXpert by Cepheid can detect 14 hrHPV types in 1 hour in a non-batch, random-access platform.^59^ However, 1 hour time-to-result after sample collection does not meet the 35 minutes requirements of our clinicians. Seely *et al.* (2023)^60^ and Chang *et al.* (2023)^61^ have recently developed isothermal assays that detect hrHPV in a clinic setting. Both assays employ workflows similar to those described in this paper, featuring affordable and user-friendly form factors, and return results in 30 and 45 minutes, respectively, making them promising assays. At this point in the test development, the work by Seely does not genotype HPV 16 and 18, the work by Chang does not detect all hrHPV types, and neither test has a sample adequacy control. Accordingly, the test proposed here provides unique technical contributions for adding a sample adequacy control, providing results fast enough for same-visit results, and incorporating cervical sampling devices in test evaluation. Additionally, the user-centered design process employed to develop a clinically relevant rapid test can serve as an example and guide for other rapid test developers.

### Nucleic acid amplification assay detects hrHPV and human DNA in water and lysate

**Fig. 2:**
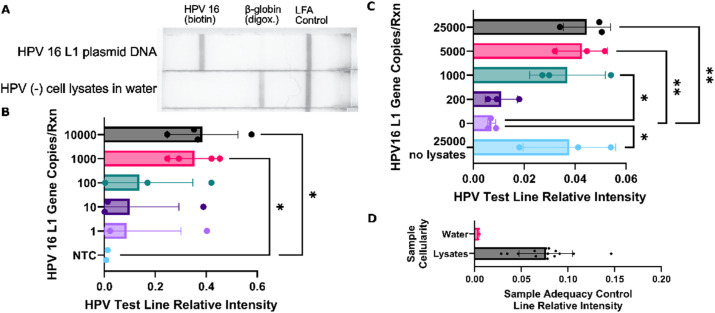
HPV 16 and β-globin detection in water. (A) Representative image of LFA readout with a line to detect HPV 16 by binding biotin-labelled amplicons, another to detect β-globin by binding digoxigenin-labelled amplicons, and a final line to verify that the LFA itself operated correctly. The LFA was generated from 500 000 copies of HPV 16 L1 plasmid DNA amplified using the same assay as in the rest of this figure. (B) The test demonstrated a LoD of 1000 for plasmid DNA bearing the HPV 16 L1 gene (*n* = 4). (C) LoD of 1000 copies per reaction demonstrated for HPV 16 L1 gene DNA in 10% reaction volume of lysates (*n* = 3). (D) The signal from the sample adequacy control of the same cell lysate samples as B compared to water controls. All error bars show standard deviation, *α > 0.05, **α > 0.01.

HPV type 16 DNA was detected at a LoD of 1000 copies per reaction in molecular biology water (Fig. 2B) and in 10% cell lysate in water (Fig. 2C). The novel β-globin specific probe provided a sample adequacy control with a detectable signal with as few as 750 copies per reaction in 10% cell lysate, low enough to detect the 1000 minimum expected genome copies of a valid cervical sample (see the SI Sample cellularity calculations section for details). To test the sample adequacy control consistency with hrHPV DNA, the probe system was run on the same water and 10% lysate samples with HPV 16 plasmid DNA used in the LoD studies. Across HPV 16 DNA concentrations, the signal from water was negligible while the signal from lysates was much stronger (Fig. 2D).

These results are consistent with the target LoD of 500–1000 copies per sample (20 000–40 000 copies per mL) of both hrHPV and human DNA for detection, even in cervical samples with the minimum required cellularity extrapolated from the Bethesda standards using the viral titer of cervical samples.^62^ Comparing our LoD to the LoD for HPV16 of other tests, Seely *et al.* reached 20–200 copies per mL (1–10 copies per swab),^60^ Chang *et al.* reports 1000 copies per mL (50 copies per swab),^61^ and COBAS HPV which is a clinical laboratory test, reaches 600 copies per mL.^63^ Further assay optimization may lower the test's LoD to be in line with these other tests, however, there is not strong evidence that a lower LoD would improve test performance and the clinical relevance of the results.^64^ Gains in sensitivity of detecting infections must be balanced with specificity and the test's ability to detect infections associated with pre-cancerous and cancerous lesions, as HPV DNA detection is used as a secondary marker of another disease state, in this case, cancer.^50,65–67^

### Clinic-amenable sample testing process

**Fig. 3:**
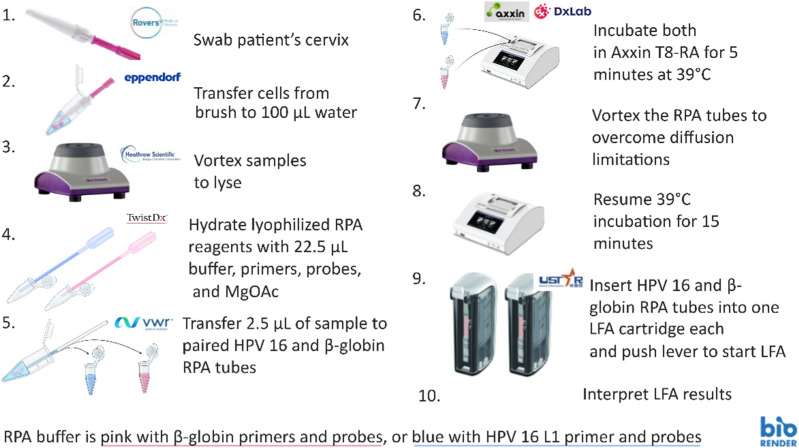
Proposed workflow for hrHPV detection in outpatient clinics. Note that step 4 was performed with micropipettes instead of exact-volume micropipettes and pre-aliquoted buffer volumes for the data presented. MgOAc is a magnesium acetate solution, a component of the TwistDx kit.

Based on our design specifications, we developed the hrHPV testing protocol in Fig. 3 for primary HPV testing of healthy patients.

First, a cervical swab is collected using the Viba Brush according to the product instructions, and the cells are then transferred to 100 µL of nuclease-free water. Following the swabbing and lysis steps, pre-aliquoted lyophilized RPA enzymes are activated with 22.5 µL of their respective liquid buffer, one reaction detecting HPV 16 and the other detecting a sample adequacy control gene, β-globin. Both reactions receive 2.5 µL of sample lysate pipetted with a plastic capillary and are run in parallel at 39 °C for 20 minutes on a heater mixer or heat block. Five (5) minutes into the incubation, the RPA solutions are removed and vortexed briefly for 5 seconds at 3200 rpm to ensure adequate mixing. Finally, each reacted RPA tube is inserted into labelled LFA cartridges (hrHPV and sample control) with a LFA strip corresponding to the hrHPV or sample control probe.

After sample collection, the process is estimated to take 35–40 minutes to obtain results (5 minutes of preparation, 20 minutes of incubation, and 10 minutes of LFA development). The process relies on commercially available equipment, with the pre-mixing of RPA reagents and the preservation of primers and probes through lyophilization or vitrification being the only manufacturing steps required to implement this test procedure in clinical applications. The technology could be further simplified in future work by replacing the Axxin T8-RA with a heating block, provided that performance remains acceptable. In either case, the use of commercially available equipment not only makes the test easier for outpatient clinics to use but also substantially reduces the number of manufacturing steps between this proof of concept and clinical implementation at scale. A bill of materials is available in supplemental materials (Table S3).

### Reproducibility of clinic-amenable sample testing process

**Fig. 4:**
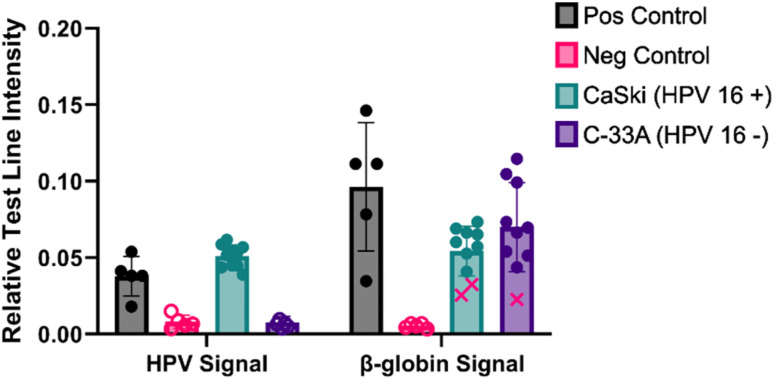
Ten replicates of C-33A (HPV 16−) and CaSki (HPV 16+) with positive and negative controls quantified at the HPV 16 and β-globin sample adequacy control LFA lines. For clarity, points that are expected to generate no signal are denoted as empty, and red Xs indicate false negative signals as determined by the limit of detection calculated from controls. All error bars show standard deviation.

To evaluate the reproducibility of the assay, 10 samples of cultured C-33A (hrHPV negative) and 10 cultured CaSki (HPV 16 positive) were tested using the technique described in Fig. 3. The LFAs were quantified and the samples were evaluated to be true/false positives and negatives based on a limit of detection calculated from the control samples (Fig. 4). For C-33A cells (HPV 16 negative), 9/10 sample adequacy control lines were positive and all 10/10 HPV 16 test lines were negative. This gives one invalid test and no false positives. For CaSki cells (HPV 16 positive), 8/10 sample adequacy control lines were positive, and 10/10 HPV test lines were positive. This gives no false negatives and no invalid samples. The interference seen in the sample adequacy control in the one negative and two positive samples does not change the interpretation of an HPV positive test, however it does suggest opportunities to further improvement of β-globin detection alongside HPV 16. There is also potential for a stronger signal from the primers and probes for HPV detection on the LFA test line, since one of the positive controls is quite close to a negative signal. Altogether, of the 20 samples tested, no false positives or negatives occurred.

### Limitations

Several project limitations warrant attention, including the paucity of patient input and reliance on self-report data from clinicians from a subset of the United States. Forthcoming information about patient preferences from our group and discordance between clinicians' reported and empiric behaviors may change the test's user requirements in future work, and limit the generalizability of our findings. Regarding the test process, the user is required to vortex the sample 5 minutes into a 20 minutes incubation. This time-sensitive, manual task poses the biggest potential interruption to the clinic workflow, and removing it with adjustments to the RPA assay would be ideal. Similarly, testing the entire process with pre-alliquoted reagents and a non-expert user would reveal potential weaknesses in the proposed clinic workflow. Another limitation is the conservative choice of targeting HPV L1 DNA, which maximizes agreement with comparator tests and minimizes test processing steps, but theoretically compromises specificity for cervical cancer risk compared to alternative biomarkers, such as mRNA of the E6 or E7 HPV gene, which would more directly indicate carcinogenic changes.^68^ Finally, only HPV 16 was targeted for this work, and it was tested in contrived samples of cultured cell lysates, without the interferents and potential inhibitors expected in patient samples. Next steps would include testing the assay on patient samples in an outpatient clinic, soliciting prototype feedback, and integration of a multiplex of hrHPV types in the next prototype iteration.

### Future work

The next step for this hrHPV rapid testing process is to evaluate the point-of-care workflow with clinical sample matrices in an outpatient clinic environment for analytical performance and user-rated test feasibility using the system usability scale.^69^ In addition to changes based on usability feedback, the next iteration of the testing process would detect other hrHPV types. In principle, this expansion would require only adding back primers of the modified PGMY11/GP6+ primer set by Gong *et al.* (2021)^33^ and designing new oligonucleotide probes for the other hrHPV types. The selection of tags on the probes would enable grouping of the hrHPV genotypes into LFA lines for HPV 16, 18, and ‘hrHPV other,’ meeting the survey and interview specifications. Finally, running the test amplicons and sample adequacy control amplicons on the same LFA would reduce the cost and steps of the test.

## Conclusions

We found that clinicians in our study were most supportive of adopting hrHPV POC testing for provider-collected cervical samples. Clinicians valued an hrHPV test that allowed for face-to-face communication of patient results during routine screenings, thereby increasing retention for follow-up treatment and care. By soliciting this input before major design decisions, the test could be made to precisely fit its demands with features such as 40 minutes sample to answer detection, sample adequacy control, and information about HPV type. The test operation and equipment required were also designed around what is or could be made available at American outpatient clinics, with only one waiting period and no watching steps to minimize clinic interruptions.

The result was a rapid testing process that meets performance standards with results available in a patient's outpatient clinic appointment, using mostly commercially available materials. Specifically, we demonstrated HPV 16 detection using a process that could be performed in an outpatient clinic by a medical assistant in under 40 minutes, allowing the results to be explained to the patient face-to-face in the same visit as the sample collection. The test has achieved a LoD of 1000 copies per reaction of HPV 16 L1 gene DNA in 10% cell lysates, and correctly identified 10 of 10 contrived HPV 16 positive and 9 of 10 contrived HPV 16 negative swabs of cultured cells, with one invalid result. These results are consistent with the performance expectations of our users and the pathology standards set in the Bethesda protocol, making the test process well poised for clinical adoption in future work. This fit-for-purpose hrHPV POCT was only possible with a user-centered design process from the device's inception.

## Author contributions

LPB, FH, ALCD, SS, and ADP contributed to laboratory experiment conceptualization, methodology, data analysis, and visualization. LPB, LAC, SS, and LNB contributed to survey and interview conceptualization, data curation, formal analysis, and visualization. ACE contributed supervision, conceptualization, and resources to the laboratory work and design specifications. NMR and JCL contributed conceptualization, resources, software, supervision, investigation, and project administration. LPB was responsible for the original manuscript draft, and all listed authors reviewed and edited the manuscript.

## Conflicts of interest

JCL is co-founder and part owner of two startup companies in portable diagnostic and monitoring technologies: Rescue Biomedical LLC, and EverTrue LLC. These are documented in an annual financial conflict of interest report that is reviewed and approved by Purdue University's Office of Research for Ethics and Compliance. The other authors declare no competing interests.

## Supplementary Material

AY-018-D5AY01621E-s001

## Data Availability

Supplementary information: an explanation of the detection and reporting mechanism, calculations for the target limit of detection, probe sequences, a bill of materials, additional experimental data from swabbing device experiments, interview protocol, and survey questionnaire items. See DOI: https://doi.org/10.1039/d5ay01621e. Full datasets are available upon reasonable request to the corresponding author.
