# Supplementary material for: Development of an HPV 16 rapid test founded in user-centered design with primary care clinicians
Source: Anal Methods. 2025 Nov 25;18(4):786–99. doi: 10.1039/d5ay01621e (PMC12679353; doi:10.1039/d5ay01621e)
Supplement: AY-018-D5AY01621E-s001 [file AY-018-D5AY01621E-s001.pdf]

Supplementary Information (SI) for Analytical Methods.
This journal is © The Royal Society of Chemistry 2025

Development of an HPV 16 Rapid Test Founded in User-Centered Design with Primary Care Clinicians - SUPPMENTAL MATERIALS

Contents

Principle of Detection and Reporting 2
DNA amplification 2
Lateral flow assay (LFA) reporting 3
Primer and probe sequences 4
Swabbing experiments 4
Background 4
Methods 5
Results 5
Sequencing Amplicons 6
Sample Cellularity Calculations 6
Expected sample cellularity and viral load 6
Required Limit of Detection 7
Table S2 User Requirements and Technical Specifications for POC HPV Test Based on Clinician-Stakeholder Input – Full Version. 9
Table S3 Bill of materials for in-clinic testing method 12
Clinician Interview Protocol 13
Selected Clinician Survey Questionnaire Items 18
Works Cited 31

Principle of Detection and Reporting

DNA amplification

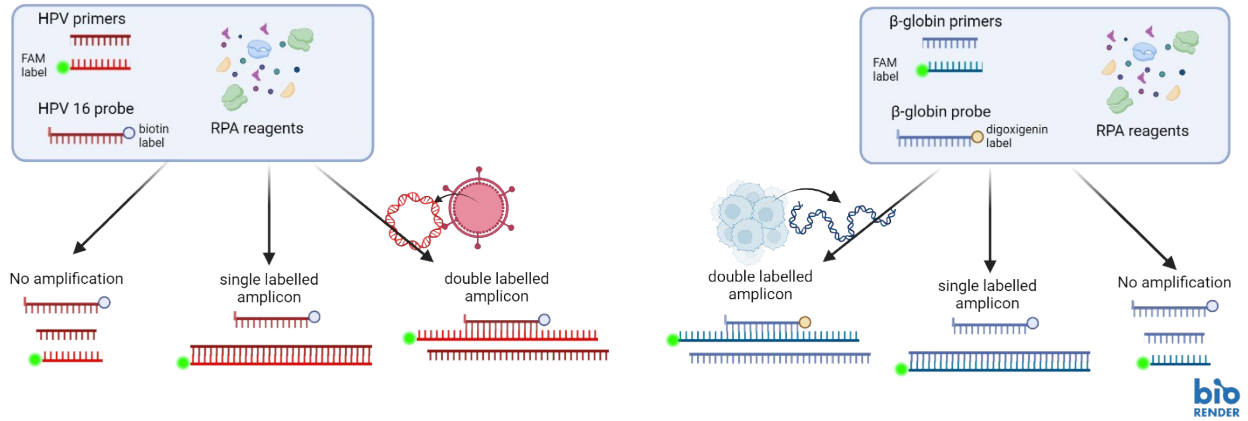

Figure S1 DNA amplification of HPV and human DNA using Recombinase Polymerase Amplification (RPA). HPV primers and probes (red) with RPA reagents can produce a double-labelled amplicon when the fluorescin (FAM) forward primer amplifies the HPV 16 L1 gene allowing the HPV probe t bind the amplicon through homologous pairing. The same is true for the β-globin primers and probes, with the biotin tag replaced with a digoxigenin tag that is captured at a test line printed with α-digoxigenin antibody.

As with most DNA amplification techniques, the sequence of interest is identified by a set of primers. In the case of HPV the forward primer is labelled with a 5' fluorescin tag. Additionally, there is a probe with homology to the amplified HPV DNA sequence between the two primers is biotin-tagged and modified at the 3' region so it cannot be extended like a primer. When the other RPA reagents, buffers, HPV 16 L1 gene DNA, and heat to 39^{oC} are introduced, this produces logistic growth. Once primers or dNTPs are exhausted, there are millions to billions of copies of the designated region of the HPV 16 L1 gene, with one strand fluorescein labelled at the 5' region, many with the biotin probe attached. The same occurs with the β-globin primers and probes with the exception that the β-globin probe While the probes competes with the complementary DNA strand, empirically there are enough amplicons with fluorescein and biotin tags at the time of detection to generate a reliable signal.

Lateral flow assay (LFA) reporting

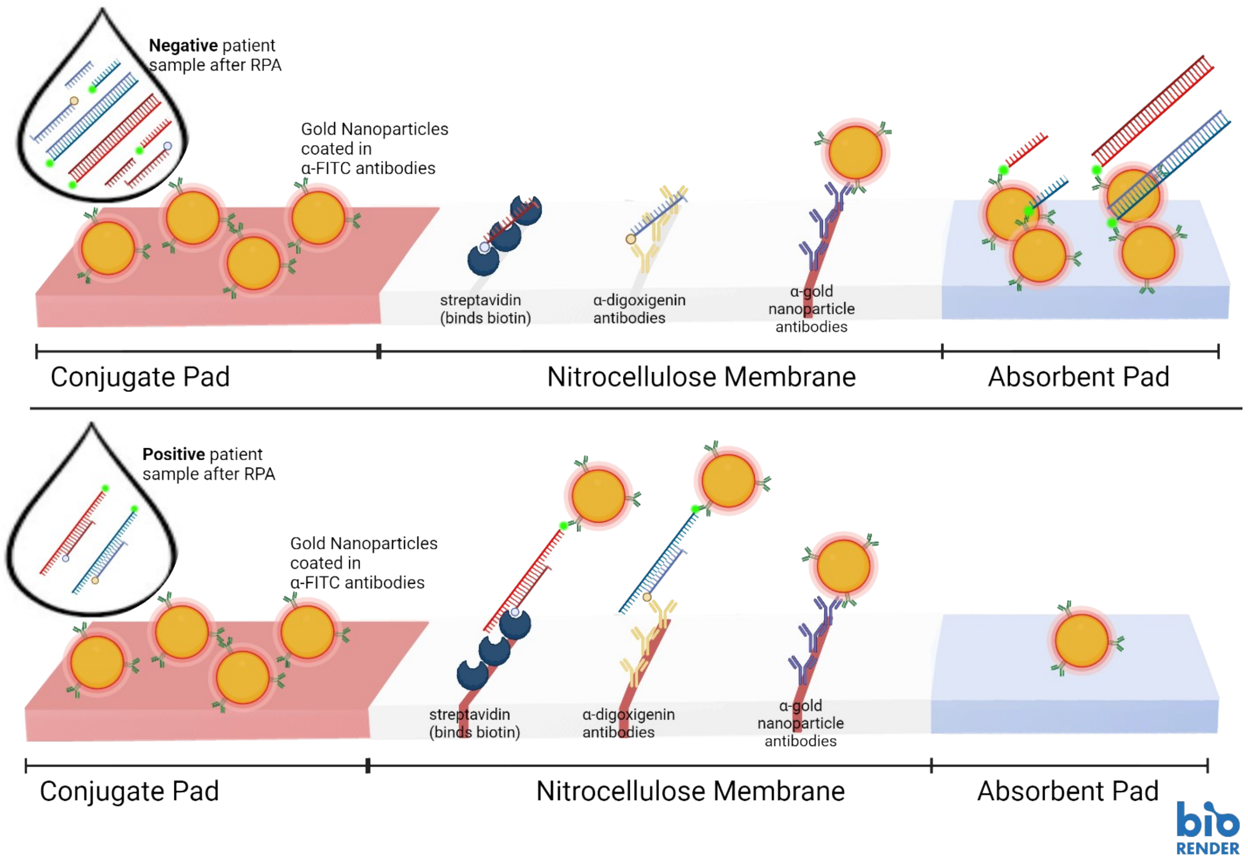

Figure S2: LFA detection of amplified DNA. Top: a negative sample of unamplified primers and probes, or products of off-target amplification (extended primer without homology to probe) will bind to the LFA test strips without anchoring the red signal-producing gold nanoparticles. Only the test line, binding directly to the gold nanoparticles, will develop the red color change. Bottom: a positive sample of amplified HPV 16 L1 DNA has amplification products with tags on both ends, anchoring gold nanoparticles to the test lines.

The LFA assay detects the tags on the amplicons to generate a red color change at printed likes on a strip of paper. Gold nanoparticles, which appear red in great number, are coated with anti-fluorescein antibodies which will strongly bind fluorescein. Similarly, the LFA has anti-gold antibodies affixed to the paper on one line, and streptavidin (which binds strongly to biotin) on the other line. When the LFA is hydrated with a sample, the gold nanoparticles are solubilized, flow down the strip, and create a red color change wherever they remain bound to a line on the LFA strip.

If the RPA reaction does not amplify DNA, or if the primers amplify DNA other than its intended target, the tagged primer and probe will not be not connected, giving no reason for the gold nanoparticles to bind at the test line and create a color chang there. However, when the RPA reaction amplifies DNA, the amplicon bearing the fluorescein tag and the homologous probe

with the biotin tag (for HPV) or digoxigenin tag (for β-globin) become effectively one molecule with both tags. When the fluorescein end binds the gold nanoparticles and then biotin end of the same molecule attaches the paper-bound streptavidin, the gold nanoparticles accumulate at the streptavidin line (aka the test line) and generate a red color change to indicate successful detection.

Primer and probe sequences

Table S1 Primer and probe sequences used in experiments.

Identifier	Sequence (5'→3')	Purpose
FAM_RPA_PGMYB	/56-FAM/GCG CAG GGC CAC AAT AAT GG	HPV forward primer based on Gong et. al.(1)
Gong_GP6+	GAA AAA TAA ACT GTA AAT CAT ATT C	HPV reverse primer based on Gong et. al.(1)
HPV16_probe	/5Biosg/GTA GTT TCT GAA GTA GAT ATG GCA GCA CAT AAT GAC /3Phos/	HPV 16-specific biotin end-labeled probe for LFS detection based on exo probe A
FAM_HBB_fwd	/56-FAM/ CTTCATCCACGTTCACCTTGCCCCACAGG	β-globin primers and probe generated by PrimedRPA, labeled to produce a signal at the digoxigenin test line on the LFS.
HBB_rev	CTGTCATCACTTAGACCTCACCCTGTGGA	
HBB_probe	/5DigN/ CTGTGTTCACTAGCAACCTCAAACAGACA /3Phos/	
*note /3Phos/ is a 3'-linked phosphate group to prevent extension. /56-FAM/ is a 5'-linked 6 fluorescein group to bind gold nanoparticles of the LFS. /5Biosg/ is a 5'-linked biotin group to attach to the first test line of the LFS. /5DigN/ is a 5'-linked digoxigenin group, which binds to the second test line on the LFS. /iFluorT/ is a 6-fluorescein group attached to a thymidine base. /idSp/ is an abasic site in the DNA, which acts as the substrate for the exonuclease once bound in a dsDNA complex. /iBHQ-1dT/ is a proximity-based quencher of 6-fluorescein.		

Swabbing experiments

Background

Cervicovaginal samples are collected with several device types, some of which have been tested for efficacy when patients use them to collect their own samples. The test proposed here is being designed for clinician-collected samples, but self-sampling amenable swabs were tested to facilitate an option for self-sampling in future work.

Methods

The Viba Brush, nylon-flocked swab, and polyester-flocked swab performed well in the literature for cervicovaginal self-sampling(2–6), and were tested for the cellularity of the samples they collect. Swabs of c33A cells at 70-80% confluence were collected in a 9 cm^2 area of a 25

cm² flask. Cells were eluted from the sampling devices into 200 µL of PBS in an Eppendorf Protein LoBind tube using standard methods(7,8). Cellularity was assessed using a trypan blue exclusion assay, scaled to account for the 4.9 cm² average human cervix[1] versus the 9 cm² flask area swabbed, and compared between swabs with a one-way ANOVA with a post-hoc Tukey's test as appropriate. In subsequent experiments a 1 cm² area was swabbed and diluted in 100 µL of fluid to minimize the culture flasks required for subsequent experiments. 2.5 µL of this fluid was added to each 25 µL RPA reaction.

Results

Each collection device needs to collect a sample with enough DNA to meet the 25,000 human genome copies of the Bethesda standard in our hands. To work with our test's LoD of 1000 copies/reaction for HPV DNA, 40,000 human genome copies/sample are required assuming a conservative 1:1 ratio of human to HPV genome copies. Of the devices tested, only VibaBrush exceeded this threshold (Fig S2).

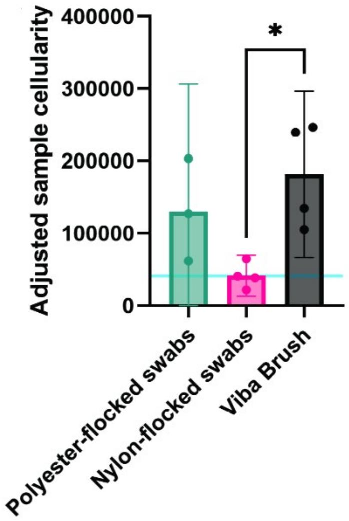

Figure S3: Cells captured from sampling devices from a 1-1.5 cm² area of flasks. The blue line represents 40,000 cells/mL, which corresponds to the assay's LoD of 1000 copies/ 25 µL. All error bars show standard deviation, * $\alpha > 0.05$, ** $\alpha > 0.01$.

Sequencing Amplicons

Standard RPA reactions were run with HPV primer/probe and β-globin primer/probe sets, followed by elution of amplicons from the test lines of LFS using a previously published method. The eluted DNA was purified and sequenced at the Purdue Genomics Core. Geneious Prime was

[1] 2.5 cm diameter(9) gives 4.9 cm²

used for sequence alignment, and primer and probe sequences were mapped to assembled sequences.

Sample Cellularity Calculations

Expected sample cellularity and viral load

Cervical cancer screening is conducted on endocervical samples, endocervical cells collected by a clinician using a swab or brush guided by a speculum. This is the sample of choice based on the results of Aim 1 below. This sample will contain endocervical (mucous-producing) cells, squamous cells (which may be metaplastic, precancerous, or otherwise irregular), as well as mucous, bacteria, viruses, immune cells, and trace blood products. The cells and HP viral load of these samples vary greatly, but between a few different estimates it seems samples generally have around 10 ng of DNA (average 13.7 ng from self-collected samples(10) and 14 ng from provider collected swabs for CIN I patients(11)). Using formula 1 these correspond to 4570 and 4667 human cell copies. Given that the cellularities presented in these studies varied by multiple orders of magnitude and little additional information about the underlying distribution was provided, clinical standards may provide a more pertinent criterion. The Bathesda guidelines give a minimum cellularity for liquid based cytology of 5,000 cells(12). Given that liquid-based cytology samples are diluted 5x in sample processing(13), original samples must contain 25,000 cells to be adequate. It also seems to be a commonly reported belief that a pap smear can be expected to contain 100,000-200,000 cells(14) though this author could not find a primary source for this quote. Accordingly, it seems safe to say that a valid cervical sample could be expected to have at least 25,000 copies (75 ng) of cellular DNA.

Equation 1

$$X\ ng\ DNA/sample\ *\ (2\ genes/copy)\ *\ (1000\ pg/ng)\ /\ (6pg\ DNA/cell)\ =\ Y\ copies/sample$$

These cell counts give an estimate of the number of HPV genome copies that could be expected from a given sample. In one study a patients with an incident HPV infection had an average viral load of log(10) 1.8/ng of cellular DNA, or 4,732 copies per sample, extrapolating from above(10). Another study estimated that over 55% of HPV 16 cases had over 1,000,000 HPV copies per sample with under 5% of patients having fewer than 100 copies per sample (conservatively extrapolated from viral titers and average cellular DNA from a normal patient sample) (11). Finally, a third study found a log(10) transformed viral load of 3.1 (1.2-8.3 95% CI) which is about 1000 viral copies per human cell copy) or higher for CIN1+ patients and 0.7 (0.3-1.5 95% CI) for normal patients, which is about 0.85 viral copies per human cell copy (15). Combining these data, a valid endocervical sample from a patient without prior diagnosed disease could conservatively be expected to have about 1:1 viral copies to human cell copies.

Combined with the cellularity data above, this gives a low confidence estimate of 25,000 HPV copies.

Required Limit of Detection

Sample dilution is bounded by 2 factors. First, RPA begins to be inhibited by background DNA of 200 ng/sample, and is completely inhibited by 0.5 ug/sample(16). Cellularity DNA varied widely across all studies as described above, however they average around 10 ng which is too low inhibition for most samples without dilution. The second bounding factor is reaction volume. Endocervical samples are most often collected by a cytological brush or a flocked swab, the latter of which can collect around 125 µL of fluid (and releases around 2/3 of this volume in our experience)(17,18).

To summarize the sample so far, at minimum 25,000 cells and around as many HPV copies are suspended in 125 µL of vaginal fluid including mucous, bacteria, and trace blood and immune cells. Around 50-80 µL of this fluid is recoverable from the sampling device at 200,000 viral copies/mL and 200,000 human cells/mL.

Realistically, an HPV molecular test of 25 µL made in an academic lab would use a patient sample comprising 10-20% of its volume. These constraints mean $1/25^{th}$ to $1/50^{th}$ of the cellular and HPV DNA present in the original sample can be tested. This gives the nucleic acid amplification test (NAAT) a target LoD from 25,000*(1/25) to 25,000 *(1/50) giving a LoD of 1,000 to 500 copies per reaction for both human and HPV DNA per reaction.

Table S2 User Requirements and Technical Specifications for POC HPV Test Based on Clinician-Stakeholder Input – Full Version.

ANALYTICAL PERFORMANCE (What quality of information is required to fulfill clinical utility)		
**User requirements**	**Design Specifications**	**Evidence/Rationale**
Perceived accuracy comparable to laboratory test standards	LoD of 1000 copies/sample(4) maintained in the presence of vaginal microbiota DNA and other interferents<br>• Under <10% false positive rate with uninfected samples.<br>• Assay demonstrates specificity to gonorrhea, chlamydia, and lactobacillus.<br>• Maintains analytical performance above these thresholds with cell lysates and vaginal mucus in sample matrix.	• Sensitivity >90% of reference test(19)<br>• Specificity >98% of reference test(19)<br>• Sensitivity >95% of lab test' and 'Specificity >95% of lab test' were the first and second most important test attributes for adoption in the clinician survey<br><br>*"The key with screening for any type of cancer is that we have an accurate result, number one, an accurate test is performed."*
Agreement with existing laboratory-based testing (HC2, Cobas HPV 4800, Aptima)	The test must demonstrate Cohen Kappa > 0.41 with Cobas HPV 4800(20) or PGMY09/11(21) on 30 HPV 16 positive and 30 HPV negative clinical samples.	Clinics will likely informally compare rapid test results with their existing HPV diagnostics: "*[a point of care test] at a previous clinic that I found it was conflicting with the lipids that I would draw through the lab. So, I didn't I've never used it here. The ones that we do use are pretty on point."*
Genotyping of high-risk strains	Test differentiates hrHPV 16, 18 positivity from positivity for all other hrHPV types.<br>• If possible, provide a separate signal for HPV 31, 33, 58<br>• If possible, separate readout for low-risk, warts-causing HPV strains HPV 6 and 11	• All clinical guidelines adopted in the United States involve separate follow-up for hrHPV 16, 18 versus hrHPV other(19,22)<br>• 'Differentiates high-risk HPV types' was the third most important test attribute for adoption in our clinician survey<br>• *"So, and some of our colposcopy guidelines are different for 16 and 18 versus other high risk, high so ideally I think if you could separate 16 and 18".*<br>• *"I think in the end, if it comes out high risk, I think that's enough for me"*<br>• HPV 33, 31, 58 were shown to have a CIN3+ risk similar to HPV 18(23,24)
Provide information about test validity (including sample adequacy and proper test function)	The test detects human DNA alongside HPV DNA. The detection of human DNA indicates that the sample was adequate for detection of HPV if present. This will be referred to as a sample adequacy control.	• *"You know how the adequacy of collection, how that can be, you know, guaranteed or assured."*<br>• *"the screening test has to be reliable. ... we've got to sample and now you're doing a confirmatory test. ... that doesn't feel good clinically, and it adds to the patient's experience you can never black or white answer it's muddy."*
If possible, provide information about other STI infections	The endocervical swab sample collection process for the HPV test described here:<br><br>• Must not prevent endocervical or vaginal swabbing for other tests in the same visit or<br>• The sample collected for hrHPV testing must leave at least half of the sample cell suspension volume unused in a medium free of chemical or molecular inhibitors that could impede detection on most rapid tests (high concentration of surfactant or chaotropic salts, soluble proteinases, very high or low pH, etc…)	*"Because I get a lot of patients who you're just here for their Pap, but they come back with trichomonas, they come back with chlamydia."*
**USABILITY**		
**User requirements**	**Design Specifications**	**Evidence/Rationale**
Be performed in outpatient clinics by a medical assistant or nurse	• CLIAA waived<br>• Does not require reagent grade water(19)<br>• Focused device training sufficient to perform test without laboratory training(19)<br>• If possible, does not require constant power supply (resource-limited settings)(19)<br>• If possible, allow samples to be run in batches as well as individually(19)	
Gives results quickly enough that a patient can be sampled and receive results in the same visit	• The test's results must be available in 40 minutes or less.	• *"It's going to have to be rapid has in five minutes or less...And if you're sitting there with a big paper towel wrapped around you. Five minutes seems like an hour."*<br><br>• *"I would say 15 minutes or less."*<br><br>• *"We run labs, I would probably say within like 20*

		minutes."
Operatable such that it does not significantly detract from other clinical duties of test operator	Recommended that the test be operable such that:<br>• Test reagents are stable at room temperature for 15 minutes before sample addition<br>• There are 5 or fewer user steps to perform**<br>• The user does not have to watch or monitor any part of the test*<br>• Test results can be communicated to a clinician in one sentence, and recorded in an electronic health record in a standardized, searchable format.	could sit at room temp for at least 15 minutes after collecting sample in case there was delay in processing the specimen.<br><br><6 steps to perform' was the 5th most important test attribute for adoption in the clinician survey a<br><br>• *"So, that was probably our biggest, our biggest issue with adopting something like that is just having someone who can consistently operate it."*<br>• *"And sometimes we're short staffed with like medical assistants, you know, and they're also doing you know pregnancy tests and HIV and, you know, drawing blood and doing other things so it would have to be pretty, pretty simple."*<br>• *"From beginning to end like opening up the package and everything... maybe four, four or five I think would be reasonable."*<br>• *"If I had to put a number, maybe 5 steps maximum."*<br>• *"For a health professional to do two or three steps, it's usually not, not too difficult."*
Cannot put a cost burden on the clinic greater than existing laboratory-based testing after reimbursement*	Reimbursed by Medicaid and major insurers such that cost to clinic is equal to or less than laboratory testing*	• *"Cost would be important to be covered by insurance or cost to be comparable to testing that exists currently. Big things."*<br>• *"... have to check that the RDT is reimbursed at least to cover costs. Including staff time.*<br>*So as soon as the rapid test became cost comparable. It was, it was readily adopted."*
Inexpensive to patients*,**	Covered by major insurers*<br><br>total costs under $24 per test to the patient after Medicare and insurance coverage.	HPV laboratory testing was estimated at over $24 per sample at cost(25)<br><br>HPV laboratory testing costs $24 or more per run and >$10,000 upfront instrument costs(26)<br><br>*"Yeah, nobody's going to walk in and lay down a $50 bill for a test like this, the test would have to be comparable in price to a pregnancy test"*
If possible, allow providers to track the HPV status of their patients*	HPV test results and test date can be integrated into existing electronic health record patient tracking systems	*"I don't remember that Mary Jane had a pap 30 days ago, and I never got the results from it. I don't have a system to remind me every pap that I collected."*
* From Indiana clinician interviews<br>** From Indiana clinician survey<br>*** From Indiana clinician interviews and surveys		

Table S3 Bill of materials for in-clinic testing method

Component	cost (USD)	runs per unit	cost per run (USD)	upfront cost (USD)
Flocked swab	$332	500	$0.66	
Rovers Viba-Brush®	$1.02	1	$1.02	
1.5 µL Eppendorf Protein LoBind® Tubes	$34	100	$0.34	
Milenia Hybridetec 2T LFS	$312	50	$6.24	

RPA kits*	$470	96	$4.90	
Mini Vortexer Heathrow Scientific®	$103	0	$0.00	$103.00
Axxin T8-RA instrument	$6500		$0.00	$6,500.00
Silicone Tubing, 2 mm ID x 4mm OD**	$11	131	$0.08	
vWR Microcaps TLC Spotting Capillaries KT764500 PA, USA	$97.54	100	$0.98	
BioUStar® BioUstar Nucleic Acid Probes Detection Device	$3	0.5	$6.00	
Total			$ 20.22	$6,603.00

*coming off patent within the year, price to change

Clinician Interview Protocol

Introductory script

"Hello, my name is ___, and I am a ___ working on a project to design and develop a rapid diagnostic test for Human papillomavirus (HPV) to increase access to cervical cancer screening among underserved populations. We are trying to learn about how providers currently screen for cervical cancer and their thoughts on a new test like this. We also want to learn the basic attributes needed in a new screening technology for it to be acceptable and helpful to providers. Interviews like this are one of the most important tools we have to gathering this information to inform the technology design process. Do you have any questions about what I just covered?"

"Have you read the consent form I sent you via email?"

[If not, read it aloud… Even if they have, review key points]

"*Do you have any questions or concerns about that?"*

[if not…] *"Great. Then I'll ask: do you agree to be in the study as an interviewee?*"

[If they verbally affirm, then begin recording and begin the interview]

Respondent Characteristics

1. Can you tell me a little bit about yourself, your organization, and your work?
   Probes:
   A. What field of medicine/specialization?
   B. What type of provider group?

2. Can you tell me about the patients/people/populations you serve?
   Probes:
   A. Do a significant portion of your patients routinely qualify for free or reduced cost screenings based on income or other socioeconomic factors?

Perspective on current screening

3. What do you think are some of the barriers to cervical cancer screening in your patient population?
   A. When you recommend HPV screening to your patients, do most of them accept your recommendation to get screened? Why or why not?
   B. What do you think would be an effective way to address these barriers in your patient population? (probe for under-screened populations)
      a. Why do you believe this would be effective?

4. Can you walk me through how you/your clinic currently screen patients for cervical cancer?

   Probes:

   A. Do you use HPV testing in your workflow?

      [If not] What is your opinion on primary HPV testing?
      [if so] **Primary HPV testing or co-testing with pap?**

   B. How did you come to use that method/test?

      What was considered in this decision? Was there a clear decision for you or your group/practice/clinic to use this method? Who made the decision?

   C. **What do you do when someone tests positive for HPV? What is the next step?**

5. How is this method of screening working?

   Probes:

   A. Good things about it? Bad things about it?
   B. How is it to perform the screening test for you and your staff? For patients?
   C. How helpful are the results of the screening test?
   D. How is the current screening workflow working for you, from arranging the visit to communicating the results to patients and arranging follow-up care when needed? How about for the patient?

6. **Are there any ways that the screening process used in your clinic could be improved?**

Perspectives on new screening methods generally

7. Have you heard of HPV self-sampling?

   [If so]

   A. What are your thoughts on this sampling method? (*probe*: for underscreened populations)
      a. In your opinion, what are the potential benefits to self-sampling?
      b. In your opinion, what are the potential limitations to self-sampling?

8. Do you think your clinic would consider adopting HPV self-sampling? Why/why not?

   [If yes]

   A. What are some challenges or limitations to adopting this method of screening?
   B. What might prevent it from being adopted?

   [If not]

   C. What, if anything, would it take for your clinic to adopt something like self-sampling for HPV?

9. What are your thoughts on self-sampling for SARS-CoV-2?

   A. Have these tests changes how you view self-sampling? How?
   B. Have these tests changed how you view self-sampling for cervical cancer? How?

10. Have you ever heard of rapid diagnostic tests?
    A. [If no]: how about point-of-care tests?
        a. [if still no, read: *A rapid diagnostic test (RDT) is a medical diagnostic test that is quick and easy to perform and typically used for preliminary medical screening or in medical facilities with limited resources. They also allow for 'point-of-care' testing in primary care settings for things that formerly only a laboratory test could measure because they provide same-day results, typically in less than an hour.]*
    B. [if yes]: Have you ever used rapid diagnostic/point-of-care test?
        a. If so, what has been your experience with RDTs?
        b. If you could think back to a time your clinic adopted a rapid diagnostic test, what was the adoption process like?
            i. How/why was the decision made?
            ii. What were the steps involved in adopting this test?

Rapid diagnostic testing for HPV
Our team of biomedical engineers is currently working on a project to design and develop a RDT that tests for the DNA of high-risk HPV types to increase access to cervical cancer screening among underserved populations. Similar to existing HPV laboratory tests, our goal is that this HPV RDT result would indicate the presence or absence of any of the carcinogenic types of HPV, but could be run on-site at the point of care, potentially within a single visit, or even in someone's home by a trained community health worker.

11. Hypothetically if this test were ready and FDA-approved tomorrow, would you consider using it in your practice/program?
    [If so]:
    A. Why would you consider using it in your practice/program?
    B. How would you use it in your screening workflow?
        a. If positive, what would your next step be?
    C. What would be the value of the RDT when used in this way? Anything else?
        a. Would this(ese) benefit(s) make it worthwhile to adopt this new test?
    D. What do you think are weaknesses or potential pitfalls, if any, to adopting this test?

    [If not], why not?
    E. What would it take for you to consider using a test like this (evidence needed? Communication channels?)

12. [for providers only] Along these same lines (hypothetically, test was ready and FDA-approved tomorrow), but for home-based HPV screening by a trained community health worker, and a patient comes in having tested positive at home, what would your next step be?
    A. Would you trust result (from home-based HPV self-sample run by CHW)? Why or why not? Or would you feel the need to repeat HPV test (using lab-based test)?

13. What would be required of an ideal RDT for you to use it in your practice/program? Specifically, do you have any thoughts on:
    A. Sensitivity/specificity requirements?
    B. What information would you need about the types? (yes/no or need to know which strain?)
    C. Ideal time to results? What is max acceptable? Why?
    D. What is the maximum acceptable number of steps/time to run the test?
    E. Who would you want/need to run the test?
    F. What materials could the test require that the user have access to? How about refrigeration, heat, or electricity?
    G. Is there any way a test would be unacceptable in how it interacts with the EHR?
    H. Cost to clinic/cost to patient?

14. [if the provider is in private practice] What would be the financial requirements of the test? Would it have to be billable? profitable? Could you tell me about the financial differences between testing performed in the office vs at home vs at the lab?
    A. Probe: What other factors might affect the reimbursement of a home-based rapid diagnostic test for HPV?

15. [For other stakeholders/non-providers]: What would be the financial requirements of the test? Feasible cost range (given no other equipment/infrastructure required)?

16. What are your thoughts on the rapid tests for SARS-CoV-2?
    A. Have these tests changed how you view rapid testing? How?
    B. Have these tests changed how you view rapid testing for cervical cancer? How?

Concluding script:
"*Those are all the questions I have for you today, thank you very much for your time. Do you have any questions or concerns before we wrap up?"* [interviewee responds]

"Thanks again for your time in this interview, and please don't hesitate to follow up with any questions, concerns, or suggestions for future study!"

Selected Clinician Survey Questionnaire Items

Start of Block: Pt. intro

Q56 Next we will ask your thoughts regarding alternative methods of cervical cancer screening. All questions relate to a 35 year-old asymptomatic patient that had a normal last screening test (normal Pap/HPV-negative) 5 years ago, like all of her previous screening tests.

End of Block: Pt. intro

Start of Block: Primary HPV Testing

Q10 Primary HPV testing is when a patient is screened for cervical cancer first using an FDA-approved laboratory-based HPV DNA test, and then **only positive cases evaluated by Pap smear**.

Q9 How familiar are you with primary HPV testing?

- This is my first time hearing of it (1)
- I am familiar with this but do not use it in my practice (2)
- I have used this in past clinical practice or training a handful of times (3)
- I currently use this occasionally (4)
- I currently use this frequently (5)

Q11 Primary HPV testing is an effective cervical cancer screening method for asymptomatic women **ages 30-65 of average-risk**.

- True (1)
- False (2)
- Uncertain (3)

Q78 To what extent do you believe primary HPV testing would improve cervical cancer screening coverage and/or follow-up for your patients?

- Greatly improve screening (1)
- Somewhat improve screening (2)
- Not improve screening (3)
- Unsure (4)

Q12 I would support adopting primary HPV testing in my practice as the preferred cervical cancer screening method **for asymptomatic average-risk women ages 30-65**:

- ○ Strongly agree (1)
- ○ Agree (2)
- ○ Uncertain (3)
- ○ Disagree (4)
- ○ Strongly disagree (5)

Display This Question:

If To what extent do you believe primary HPV testing would improve cervical cancer screening coverag... = Greatly improve screening

Or To what extent do you believe primary HPV testing would improve cervical cancer screening coverag... = Somewhat improve screening

And If

I would support adopting primary HPV testing in my practice as the preferred cervical cancer scre... = Strongly disagree

Or I would support adopting primary HPV testing in my practice as the preferred cervical cancer scre... = Disagree

Q79 Why wouldn't you support this method of screening?

____

End of Block: Primary HPV Testing

Start of Block: Point-of-Care Testing

Q73 *All questions relate to a 35 year-old asymptomatic patient that had a normal last screening test (normal Pap/HPV-negative) 5 years ago, like all of her previous screening tests.*

Q13 Point-of-care tests are rapid diagnostic tests performed and analyzed by a medical provider during a patient's clinic visit.

Q14 How familiar are you with point-of-care testing, for any purpose?

- This is my first time hearing of it (1)
- I am familiar with this but do not use it in my practice (2)
- I have used this in past clinical practice or training a handful of times (3)
- I currently use this occasionally (4)
- I currently use this frequently (5)

Q15 A number of point-of-care tests are in development and being evaluated for HPV screening. These would work very similarly to existing point-of-care tests (for example, rapid HIV or rapid flu tests) and would allow providers to determine if a patient was high-risk HPV positive within minutes, while the patient is still in the clinic. This is called a point-of-care HPV test.

Q16 To what extent do you believe point-of-care HPV testing would improve cervical cancer screening coverage and/or follow-up for your patients?

- Greatly improve screening (1)
- Somewhat improve screening (2)
- Not improve screening (3)
- Unsure (4)

Q17 Assuming this test was FDA approved, I would support adopting point-of-care HPV testing in my clinic:

- Strongly agree (1)
- Agree (2)
- Uncertain (3)
- Disagree (4)
- Strongly disagree (5)

Display This Question:

If To what extent do you believe point-of-care HPV testing would improve cervical cancer screening c... = Greatly improve screening

Or To what extent do you believe point-of-care HPV testing would improve cervical cancer screening c... = Somewhat improve screening

And If

Assuming this test was FDA approved, I would support adopting point-of-care HPV testing in my cli... = Disagree

Or Assuming this test was FDA approved, I would support adopting point-of-care HPV testing in my cli... = Strongly disagree

Q63 Why wouldn't you support this method of screening?

____

End of Block: Point-of-Care Testing

Start of Block: Self-Sampling

Q74 *All questions relate to a* *35 year-old asymptomatic patient* *that had a normal last screening test (normal Pap/HPV-negative) 5 years ago, like all of her previous screening tests.*

Q18 Self-sampling for cervical cancer screening allows women to collect their own vaginal swab in private with an FDA-approved self-collection device and instructions. The self-collected samples can be collected in the home, workplace, or elsewhere and then sent to the laboratory by dropping it off in their mailbox, at the clinic, or given to a community health worker.

Q19 How familiar are you with patient self-sampling for any purpose, not just HPV?

- This is my first time hearing of it (1)
- I am familiar with this but do not use it in my practice (2)
- I have used this in past clinical practice or training a handful of times (3)
- I currently use this occasionally (4)
- I currently use this frequently (5)

Q20 To what extent do you believe HPV self-sampling in the clinic would improve cervical cancer screening coverage and/or follow-up for your patients?

- o Greatly improve screening (1)
- o Somewhat improve screening (2)
- o Not improve screening (3)
- o Unsure (4)

Q21 I would support offering HPV self-sampling to my patients, where a woman collects her own specimen in the clinic:

- o Strongly agree (1)
- o Agree (2)
- o Uncertain (3)
- o Disagree (4)
- o Strongly disagree (5)

Display This Question:

If To what extent do you believe HPV self-sampling in the clinic would improve cervical cancer scree... = Greatly improve screening

Or To what extent do you believe HPV self-sampling in the clinic would improve cervical cancer scree... = Somewhat improve screening

And If

I would support offering HPV self-sampling to my patients, where a woman collects her own specime... = Disagree

Or I would support offering HPV self-sampling to my patients, where a woman collects her own specime... = Strongly disagree

Q62 Why wouldn't you support this method of screening?

____

Q22 To what extent do you believe HPV self-sampling at home would improve cervical cancer screening coverage and/or follow-up for your patients?

- o Greatly improve screening (1)
- o Somewhat improve screening (2)
- o Not improve screening (3)
- o Unsure (4)

Q23 I would support offering HPV self-sampling to my patients, where a woman collects her own specimen at home without needing to come to the clinic if the results are normal:

- Strongly agree (1)
- Agree (2)
- Uncertain (3)
- Disagree (4)
- Strongly disagree (5)

Display This Question:

If To what extent do you believe HPV self-sampling at home would improve cervical cancer screening c... = Greatly improve screening

Or To what extent do you believe HPV self-sampling at home would improve cervical cancer screening c... = Somewhat improve screening

And If

I would support offering HPV self-sampling to my patients, where a woman collects her own specime... = Disagree

Or I would support offering HPV self-sampling to my patients, where a woman collects her own specime... = Strongly disagree

Q61 Why wouldn't you support this method of screening?

____

End of Block: Self-Sampling

Start of Block: At-home HPV Rapid Testing

Q75 *All questions relate to a 35 year-old asymptomatic patient that had a normal last screening test (normal Pap/HPV-negative) 5 years ago, like all of her previous screening tests.*

Q24 An HPV rapid diagnostic test like those being developed for point-of-care testing could also enable at-home testing if they were simple enough for patients to use (other examples of at-home testing include pregnancy tests and blood glucose tests). This would be called an at-home rapid HPV test. As opposed to self-sampling at home, at-home rapid HPV tests would also deliver results at home within a matter of minutes.

Q25 How familiar are you with at-home testing in general, for any purpose?

- o This is my first time hearing of it (4)
- o I am familiar with this but do not use it in my practice (5)
- o I have used this in past clinical practice or training a handful of times (6)
- o I currently use this occasionally (7)
- o I currently use this frequently (8)

Q31 To what extent do you believe at-home rapid HPV testing would improve cervical cancer screening coverage and/or follow-up for your patients?

- o Greatly improve screening (1)
- o Somewhat improve screening (2)
- o Not improve screening (3)
- o Unsure (4)

Q32 I would support offering at-home rapid HPV testing for my patients to complete without needing to come into the clinic if the results are normal:

- o Strongly agree (1)
- o Agree (2)
- o Uncertain (3)
- o Disagree (4)
- o Strongly disagree (5)

Display This Question:

If To what extent do you believe at-home rapid HPV testing would improve cervical cancer screening c... = Greatly improve screening

Or To what extent do you believe at-home rapid HPV testing would improve cervical cancer screening c... = Somewhat improve screening

And If

I would support offering at-home rapid HPV testing for my patients to complete without needing to... = Disagree

Or I would support offering at-home rapid HPV testing for my patients to complete without needing to... = Strongly disagree

Q64 Why wouldn't you support this method of screening?

____

End of Block: At-home HPV Rapid Testing

Start of Block: CHW-delivered At-home Testing

Q76 *All questions relate to a* *35 year-old asymptomatic patient* *that had a normal last screening test (normal Pap/HPV-negative) 5 years ago, like all of her previous screening tests.*

Q29 Patients could also be screened for cervical cancer at home by a specially trained community health worker (CHW). The CHW would bring the self-sampling device and an HPV rapid test to a patient's home, the patient would collect her own specimen, the CHW would perform the rapid HPV test immediately and explain the results to the patient, then navigate women who test positive to the clinic for follow-up and communicate their results to the provider. This would be called a CHW-delivered HPV test.

Q30 How familiar are you with Community Health Workers, in general, for any purpose?

- This is my first time hearing of community health workers (1)
- I am familiar with this but it is not a part of my practice (2)
- I have worked with community health workers a handful of times in the past (3)
- I work with community health workers occasionally (4)
- I work with community health workers frequently (5)

Q33 To what extent do you believe CHW-delivered HPV testing would improve cervical cancer screening coverage and/or follow-up for your patients?

- Greatly improve screening (1)
- Somewhat improve screening (2)
- Not improve screening (3)
- Unsure (4)

Q34 I would support offering CHW-delivered HPV testing for my patients to complete without needing to come into the clinic if the results are normal:

- Strongly agree (1)
- Agree (2)
- Uncertain (3)
- Disagree (4)
- Strongly disagree (5)

Display This Question:

If To what extent do you believe CHW-delivered HPV testing would improve cervical cancer screening c... = Greatly improve screening

Or To what extent do you believe CHW-delivered HPV testing would improve cervical cancer screening c... = Somewhat improve screening

And If

I would support offering CHW-delivered HPV testing for my patients to complete without needing to... = Strongly disagree

Or I would support offering CHW-delivered HPV testing for my patients to complete without needing to... = Disagree

Q60 Why wouldn't you support this method of screening?

____

End of Block: CHW-delivered At-home Testing

Start of Block: HPV Test Attributes

Q88 *These questions relate to a 35 year-old asymptomatic patient that had a normal last screening test (normal Pap/HPV-negative) 5 years ago, like all of her previous screening tests.*

Q26 Below are possible characteristics of rapid HPV tests. Assuming the test is FDA approved, how important is each characteristic for you to consider adopting rapid HPV testing for your patients (for tests performed at home or in clinic)?

	Necessary for adoption (1)	Would facilitate adoption (2)	Neutral/ Uncertain (3)	Not necessary for adoption (4)	Would prevent adoption (5)
Differentiates between types of high-risk/carcinogenic HPV strains (1)	o	o	o	o	o
Detects HPV strains responsible for genital warts in addition to the cancer-causing strains (2)	o	o	o	o	o
Test involves no more than 5 steps to perform (3)	o	o	o	o	o
Test must take less than 5 minutes to perform (14)	o	o	o	o	o
In-clinic test results are ready in less than 15 minutes (4)	o	o	o	o	o
In-clinic test results are ready in greater than 15 minutes but less than 2 hours (5)	o	o	o	o	o
Sensitivity is at least 80% of the laboratory reference test (6)	o	o	o	o	o
Sensitivity is at least 95% of the laboratory reference test (7)	o	o	o	o	o
Specificity is at least 80% of the laboratory reference test (8)	o	o	o	o	o
Specificity is at least 95% of the	o	o	o	o	o

laboratory reference test (9)					
It is made by a company I recognize (10)	o	o	o	o	o
It would generate revenue for the clinic (11)	o	o	o	o	o
It does not cost more to the patient than the existing test (12)	o	o	o	o	o

Q79 Are there any other test characteristics that are important to you?

____

End of Block: HPV Test Attributes

Works Cited

1. Gong J, Zhang G, Wang W, Liang L, Li Q, Liu M, et al. A simple and rapid diagnostic method for 13 types of high-risk human papillomavirus (HR-HPV) detection using CRISPR-Cas12a technology. Sci Rep [Internet]. 2021 Jun 17 [cited 2021 Aug 18];11(1):12800. Available from: https://www.nature.com/articles/s41598-021-92329-2

2. Viviano M, Willame A, Cohen M, Benski AC, Catarino R, Wuillemin C, et al. A comparison of cotton and flocked swabs for vaginal self-sample collection. Int J Womens Health. 2018;10:229–36.

3. Coorevits L, Traen A, Bingé L, Van Dorpe J, Praet M, Boelens J, et al. Are vaginal swabs comparable to cervical smears for human papillomavirus DNA testing? J Gynecol Oncol. 2018 Jan;29(1):e8.

4. Berggrund M, Gustavsson I, Aarnio R, Hedlund-Lindberg J, Sanner K, Wikström I, et al. HPV viral load in self-collected vaginal fluid samples as predictor for presence of cervical intraepithelial neoplasia. Virol J [Internet]. 2019 Nov 27 [cited 2021 Oct 13];16(1):146. Available from: https://doi.org/10.1186/s12985-019-1253-2

5. Vassilakos P, Catarino R, Bougel S, Munoz M, Benski C, Meyer-Hamme U, et al. Use of swabs for dry collection of self-samples to detect human papillomavirus among Malagasy women. Infect Agent Cancer [Internet]. 2016 Mar 17 [cited 2021 Oct 13];11:13. Available from: https://www.ncbi.nlm.nih.gov/pmc/articles/PMC4794859/

6. Labani S, Asthana S. Human papillomavirus viral load on careHPV testing of self-collected vaginal samples vs. clinician-collected cervical samples. Eur J Obstet Gynecol Reprod Biol [Internet]. 2014 Oct 1 [cited 2021 Oct 13];181:233–9. Available from: https://www.sciencedirect.com/science/article/pii/S030121151400428X

7. Panpradist N, Toley BJ, Zhang X, Byrnes S, Buser JR, Englund JA, et al. Swab Sample Transfer for Point-Of-Care Diagnostics: Characterization of Swab Types and Manual Agitation Methods. PLOS ONE [Internet]. 2014 Sep 2 [cited 2024 Feb 10];9(9):e105786. Available from: https://journals.plos.org/plosone/article?id=10.1371/journal.pone.0105786

8. Evalyn® Brush [Internet]. Rovers Medical Devices. [cited 2023 Jan 11]. Available from: https://www.roversmedicaldevices.com/cell-sampling-devices/evalyn-brush/

9. Kurman RJ, editor. Blaustein's Pathology of the Female Genital Tract [Internet]. New York, NY: Springer; 1994 [cited 2024 Feb 13]. Available from: https://link.springer.com/10.1007/978-1-4757-3889-6

10. Winer RL, Xi LF, Shen Z, Stern JE, Newman L, Feng Q, et al. Viral load and short-term natural history of type-specific oncogenic human papillomavirus infections in a high-risk cohort of midadult women. Int J Cancer [Internet]. 2014 [cited 2021 Oct 13];134(8):1889–98. Available from: http://onlinelibrary.wiley.com/doi/abs/10.1002/ijc.28509

11. Swan DC, Tucker RA, Tortolero-Luna G, Mitchell MF, Wideroff L, Unger ER, et al. Human Papillomavirus (HPV) DNA Copy Number Is Dependent on Grade of Cervical Disease and HPV Type. J Clin Microbiol [Internet]. 1999 Apr [cited 2024 Jan 22];37(4):1030–4. Available from: https://journals.asm.org/doi/10.1128/jcm.37.4.1030-1034.1999

12. Pangarkar MA. The Bethesda System for reporting cervical cytology. CytoJournal [Internet]. 2022 Apr 30 [cited 2024 Jan 22];19:28. Available from: https://www.ncbi.nlm.nih.gov/pmc/articles/PMC9168399/

13. Makde MM, Sathawane P. Liquid-based cytology: Technical aspects. CytoJournal [Internet]. 2022 Jun 14 [cited 2024 Jan 22];19:41. Available from: https://www.ncbi.nlm.nih.gov/pmc/articles/PMC9345114/

14. Bengtsson E, Malm P. Screening for Cervical Cancer Using Automated Analysis of PAP-Smears. Comput Math Methods Med [Internet]. 2014 Mar 20 [cited 2023 Jun 14];2014:e842037. Available from: https://www.hindawi.com/journals/cmmm/2014/842037/

15. Malagón T, Louvanto K, Ramanakumar AV, Koushik A, Coutlée F, Franco EL. Viral load of human papillomavirus types 16/18/31/33/45 as a predictor of cervical intraepithelial neoplasia and cancer by age. Gynecol Oncol [Internet]. 2019 Nov 1 [cited 2021 Oct 13];155(2):245–53. Available from: https://www.sciencedirect.com/science/article/pii/S0090825819315306

16. Rohrman B, Richards-Kortum R. Inhibition of Recombinase Polymerase Amplification by Background DNA: A Lateral Flow-Based Method for Enriching Target DNA. Anal Chem [Internet]. 2015 Feb 3 [cited 2023 Sep 13];87(3):1963–7. Available from: https://pubs.acs.org/doi/10.1021/ac504365v

17. Zasada AA, Zacharczuk K, Woźnica K, Główka M, Ziółkowski R, Malinowska E. The influence of a swab type on the results of point-of-care tests. AMB Express [Internet]. 2020 Mar 12 [cited 2021 Nov 15];10:46. Available from: https://www.ncbi.nlm.nih.gov/pmc/articles/PMC7067933/

18. Warnke P, Warning L, Podbielski A. Some Are More Equal - A Comparative Study on Swab Uptake and Release of Bacterial Suspensions. PLoS ONE [Internet]. 2014 Jul 10 [cited 2021 Nov 15];9(7):e102215. Available from: https://www.ncbi.nlm.nih.gov/pmc/articles/PMC4092111/

19. Organization WH. WHO technical guidance and specifications of medical devices for screening and treatment of precancerous lesions in the prevention of cervical cancer [Internet]. World Health Organization; 2020 [cited 2021 Jun 25]. Available from: https://apps.who.int/iris/handle/10665/331698

20. Landis JR, Koch GG. The Measurement of Observer Agreement for Categorical Data. Biometrics [Internet]. 1977 [cited 2023 Nov 21];33(1):159–74. Available from: https://www.jstor.org/stable/2529310

21. Gravitt PE, Peyton CL, Alessi TQ, Wheeler CM, Coutlée F, Hildesheim A, et al. Improved amplification of genital human papillomaviruses. J Clin Microbiol. 2000 Jan;38(1):357–61.

22. Snijders PJF, Verhoef VMJ, Arbyn M, Ogilvie G, Minozzi S, Banzi R, et al. High-risk HPV testing on self-sampled *versus* clinician-collected specimens: A review on the clinical accuracy and impact on population attendance in cervical cancer screening. Int J Cancer [Internet]. 2013 May 15 [cited 2021 Aug 8];132(10):2223–36. Available from: https://onlinelibrary.wiley.com/doi/10.1002/ijc.27790

23. Schiffman M, Vaughan LM, Raine-Bennett TR, Castle PE, Katki HA, Gage JC, et al. A study of HPV typing for the management of HPV-positive ASC-US cervical cytologic results. Gynecol Oncol. 2015 Sep;138(3):573–8.

24. Schiffman M, Hyun N, Raine-Bennett TR, Katki H, Fetterman B, Gage JC, et al. A cohort study of cervical screening using partial HPV typing and cytology triage. Int J Cancer. 2016 Dec 1;139(11):2606–15.

25. Zamani M, Robson JM, Fan A, Bono MS, Furst AL, Klapperich CM. Electrochemical Strategy for Low-Cost Viral Detection. ACS Cent Sci [Internet]. 2021 Jun 23 [cited 2021 Aug 8];7(6):963–72. Available from: https://pubs.acs.org/doi/10.1021/acscentsci.1c00186

26. Kundrod KA, Smith CA, Hunt B, Schwarz RA, Schmeler K, Richards-Kortum R. Advances in technologies for cervical cancer detection in low-resource settings. Expert Rev Mol Diagn [Internet]. 2019 Aug 3 [cited 2023 Nov 21];19(8):695–714. Available from: https://www.tandfonline.com/doi/full/10.1080/14737159.2019.1648213
